# Supplementary material for: Beyond Equilibrium Ensembles: Time Rescaling in Coarse-Grained Simulations across Single-Molecule and Condensate Regimes
Source: bioRxiv. 2026 Sep 2:2026.09.01.748708. Preprint. [Version 1] doi: 10.64898/2026.09.01.748708 (PMC13684552; doi:10.64898/2026.09.01.748708)
Supplement: Supplementary file 1 [file media-1.pdf]

Beyond Equilibrium Ensembles: Time Rescaling in Coarse-Grained Simulations across Single-Molecule and Condensate Regimes

Soundhararajan Gopi[1,*], Hanling Qin[1,3], Robert B. Best[2,*], Benjamin Schuler[1,3,*]

[1] Department of Biochemistry, University of Zurich, Winterthurerstrasse 190, 8057 Zurich, Switzerland

[2] Laboratory of Chemical Physics, National Institute of Diabetes and Digestive and Kidney Diseases, National Institutes of Health, Bethesda, MD, USA

[3] Department of Physics, University of Zurich, Winterthurerstrasse 190, 8057 Zurich, Switzerland

*e-mail: s.gopi@bioc.uzh.ch, robert.best2@nih.gov, schuler@bioc.uzh.ch

ProTα	PSDAAVDTSSEITTKDLKEKKEVVEEAENGRDAPANGNAENEENGEQEADNEVDEEEEEGGEEEEEEEEGDGEEEDGDEDEEAESATGKRAAEDDEDDDVDTKKQKTDEDD
H1	CTENSTSAPAAKPKRAKASKKSTDHPKYSDMIVAAIQAEKNRAGSSRQSIQKYIKSHYKVGENADSQIKLSIKRLVTTGVLKQTKGVGASGSFRLAKSDEPKKSVAFKKTKKEIKKVATPKKASKPKKAASKAPTKKPKATPVKKAKKKLAATPKKAKKPKTVKAKPVKASKPKKAKPVKPKAKSSAKRAGKKKGGPR
Protamine	MPRRRRSSSRPVRRRRRPRVSRRRRRRGGRRRR
Poly-L-lysine 50 (K50)	KKKKKKKKKKKKKKKKKKKKKKKKKKKKKKKKKKKKKKKKKKKKKKKKKK
Poly-L-arginine 50 (R50)	RRRRRRRRRRRRRRRRRRRRRRRRRRRRRRRRRRRRRRRRRRRRRRRRRR

Table S1. Amino acid sequences of polypeptides used in simulations.

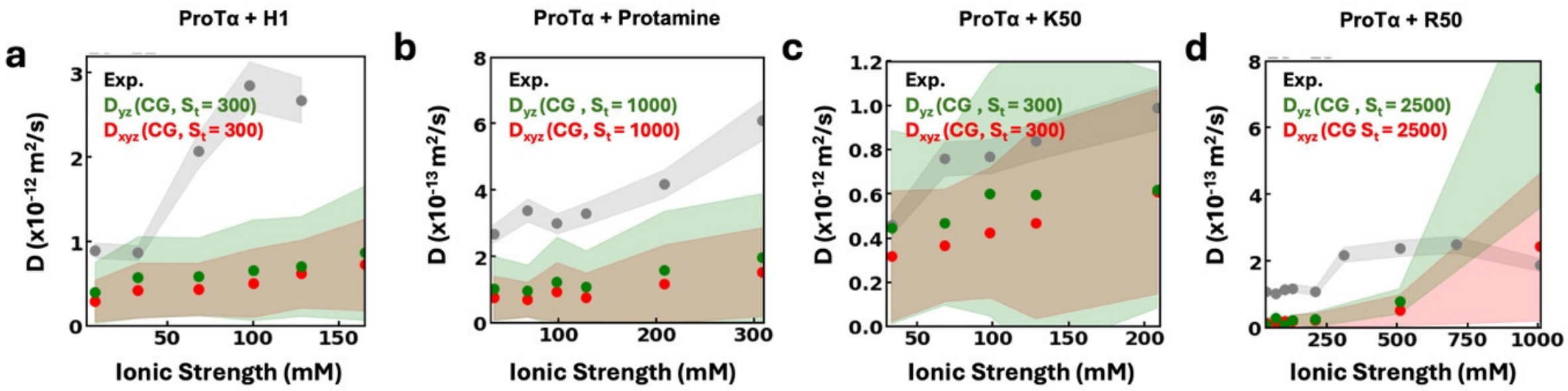

FIG. S1. Diffusion coefficients D are shown as a function of ionic strength for complex coacervates of (a) ProTα + H1, (b) ProTα + protamine, (c) ProTα + K50, and (d) ProTα + R50. Experimental diffusion coefficients[1] are shown in gray, while coarse-grained (CG) estimates obtained from the full three-dimensional displacement (D_{xyz}) are shown in red. Green symbols indicate the in-plane diffusion coefficient calculated (D_{yz}) from motion in the *yz* plane. Shaded regions indicate the corresponding uncertainty ranges. The time-rescaling factor, $S_t = \tau_{\text{exp}}/\tau_{\text{CG}}$, is specified in the figure legends.

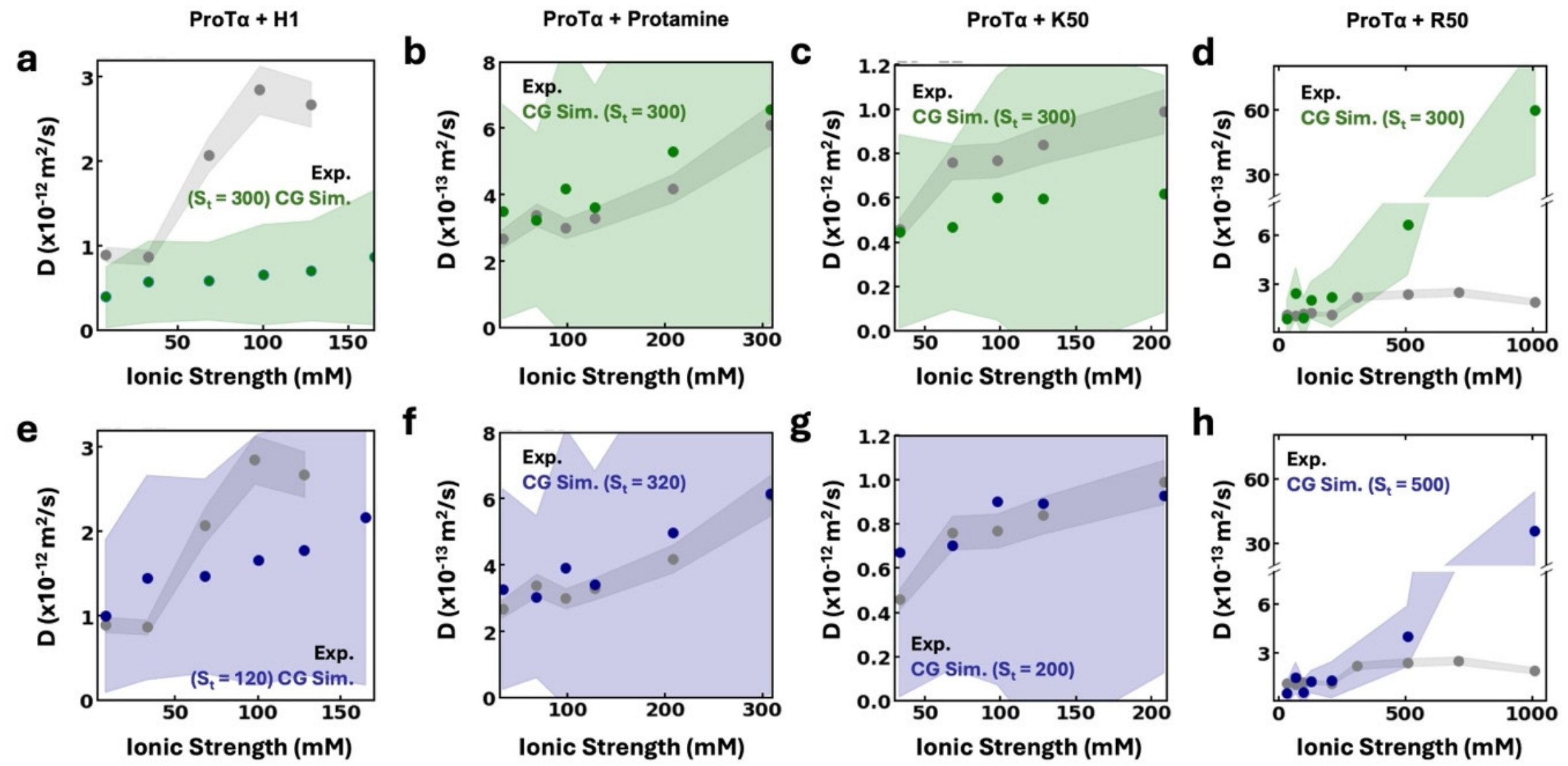

FIG. S2. Comparison of diffusion coefficient D as a function of ionic strength for the same systems as in Fig. S1 estimated from the CG simulations (green), along with the data from single-molecule experiments[1] (gray), assuming the same time rescaling factor for all simulations (a-d) and using a system-dependent time rescaling factor (blue) to maximize the agreement with experimental data (e-h). The time-rescaling factor, $S_t = \tau_{\mathrm{exp}}/\tau_{\mathrm{CG}}$, is specified in the figure legends.

References

[1] N. Galvanetto, M.T. Ivanović, S.A. Del Grosso, A. Chowdhury, A. Sottini, D. Nettels, R.B. Best, and B. Schuler, "Material properties of biomolecular condensates emerge from nanoscale dynamics," Proceedings of the National Academy of Sciences **122**(23), e2424135122 (2025).
